# The Kynurenic Acid Analog SZR72 Enhances Neuronal Activity after Asphyxia but Is Not Neuroprotective in a Translational Model of Neonatal Hypoxic Ischemic Encephalopathy

**DOI:** 10.3390/ijms22094822

**Published:** 2021-05-01

**Authors:** Viktória Kovács, Gábor Remzső, Tímea Körmöczi, Róbert Berkecz, Valéria Tóth-Szűki, Andrea Pénzes, László Vécsei, Ferenc Domoki

**Affiliations:** 1 Department of Physiology, Faculty of Medicine, University of Szeged, 6720 Szeged, Hungary; kovacs.viktoria.1@med.u-szeged.hu (V.K.); remzso.gabor@med.u-szeged.hu (G.R.); toth-szuki.valeria@med.u-szeged.hu (V.T.-S.); penzes.andrea@med.u-szeged.hu (A.P.); 2 Institute of Pharmaceutical Analysis, Interdisciplinary Excellence Center, University of Szeged, 6720 Szeged, Hungary; kormoczi.timi@gmail.com (T.K.); berkecz.robert@szte.hu (R.B.); 3 Department of Neurology, Interdisciplinary Excellence Center, University of Szeged, 6720 Szeged, Hungary; vecsei.laszlo@med.u-szeged.hu; 4 MTA-SZTE Neuroscience Research Group, Hungarian Academy of Sciences, University of Szeged, 6720 Szeged, Hungary

**Keywords:** birth asphyxia, neonatal encephalopathy, newborn pig, therapeutic hypothermia, kynurenine

## Abstract

Hypoxic–ischemic encephalopathy (HIE) remains to be a major cause of long-term neurodevelopmental deficits in term neonates. Hypothermia offers partial neuroprotection warranting research for additional therapies. Kynurenic acid (KYNA), an endogenous product of tryptophan metabolism, was previously shown to be beneficial in rat HIE models. We sought to determine if the KYNA analog SZR72 would afford neuroprotection in piglets. After severe asphyxia (pHa = 6.83 ± 0.02, ΔBE = −17.6 ± 1.2 mmol/L, mean ± SEM), anesthetized piglets were assigned to vehicle-treated (VEH), SZR72-treated (SZR72), or hypothermia-treated (HT) groups (*n* = 6, 6, 6; Tcore = 38.5, 38.5, 33.5 °C, respectively). Compared to VEH, serum KYNA levels were elevated, recovery of EEG was faster, and EEG power spectral density values were higher at 24 h in the SZR72 group. However, instantaneous entropy indicating EEG signal complexity, depression of the visual evoked potential (VEP), and the significant neuronal damage observed in the neocortex, the putamen, and the CA1 hippocampal field were similar in these groups. In the caudate nucleus and the CA3 hippocampal field, neuronal damage was even more severe in the SZR72 group. The HT group showed the best preservation of EEG complexity, VEP, and neuronal integrity in all examined brain regions. In summary, SZR72 appears to enhance neuronal activity after asphyxia but does not ameliorate early neuronal damage in this HIE model.

## 1. Introduction

Hypoxic–ischemic encephalopathy (HIE) is a devastating condition of term neonates. Its diagnosis relies on the detection of signs of birth asphyxia and the subsequent development of encephalopathy marked by altered consciousness, abnormal/absent reflexes, and alterations in brain electrical activity [1]. Severe HIE results in death or severe long-term neurodevelopmental deficits in over a million neonates yearly all over the world making a severe socioeconomic burden, in fact, HIE represents 2.4% of the total burden of disease amounting to 50.2 million disability-adjusted life years worldwide [2,3]. Currently, mild whole-body hypothermia is being employed as the only clinically effective neuroprotective therapy of HIE. However, the meta-analysis of 11 clinical trials found that the number needed to treat to benefit was 7 (range 4–17); thus, seven HIE patients with severe encephalopathy on average must be cooled to avoid one death or major disability in the population [4]. Therefore, the development of adjunct neuroprotective therapies complementing the effect of hypothermia is clearly warranted and requires continuing research effort.

Kynurenic acid (KYNA), an endogenous molecule produced in the kynurenine pathway of tryptophan metabolism, has been long known to exert neuroprotection in various preclinical models of hypoxic–ischemic brain injury including stroke, for recent reviews see [5,6]. Both post-insult administration of KYNA or combination of kynurenine with probenecid to further elevate cerebral KYNA levels were reported to also reduce hypoxic–ischemic damage in neonatal rats using the Rice–Vanucci model [7,8], but the possible neuroprotective action of KYNA in a translational large animal (e.g., newborn pig) model has not yet been tested. However, KYNA has been shown to antagonize NMDA-induced pial arteriolar vasodilation in piglets [9]. NMDA elicits dilation of cerebrocortical arterioles indirectly, through activation of neuronal NMDA receptors [10], which indicates that KYNA would antagonize neuronal NMDA receptors in this species as well, and it may exert an anti-excitotoxic effect during HIE development.

SZR72 (2-(2-N, N-dimethylaminoethylamine-1-carbonyl)-1H-quinolin-4-one hydrochloride) is a synthetic analog of KYNA developed to enhance the pharmacokinetic characteristics of KYNA [11]. SZR72 has been found to exert neuroprotective effects in a number of rat studies on nitroglycerine-induced migraine [12], neurogenic inflammation [13], and global cerebral ischemia [14]. The major purpose of the present study was to test if the post-insult administration of SZR72 would convey neuroprotection in the subacute phase of HIE development in a translational large animal HIE model, the newborn pig. The effect of SZR72-treated (SZR72 group) was compared to both vehicle-treated (VEH group) and hypothermia-treated (HT group) serving as negative and positive controls, respectively. The major study outcome measures were alterations in brain electrical activity (EEG), visual evoked potential (VEP), and neuropathology assessment.

## 2. Results

**Figure 1:**
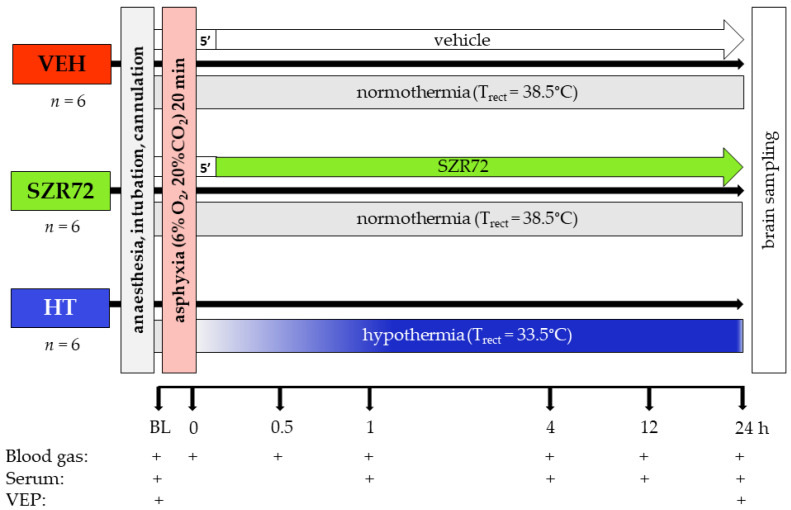
Graphic representation of the experimental protocol. Anesthetized, instrumented piglets were assigned to either the vehicle-treated (VEH), SZR72-treated (SZR72), or the hypothermia-treated (HT) groups. After obtaining baseline (BL) blood gases, serum samples, and visual evoked potential (VEP) recordings, the animals were exposed to asphyxia induced by ventilation with a hypoxic/hypercapnic gas mixture for 20 min. SZR72 or vehicle administration started 5 min upon completion of asphyxia, whereas in the HT group cooling started simultaneously with reoxygenation, rectal temperature (T_rect_) reached 33.5 °C in 40–50 min. Blood gases, serum samples, and VEP recordings were obtained at the indicated time points. At the end of the observation period, the brains were processed for neuropathology examination.

**Figure 2:**
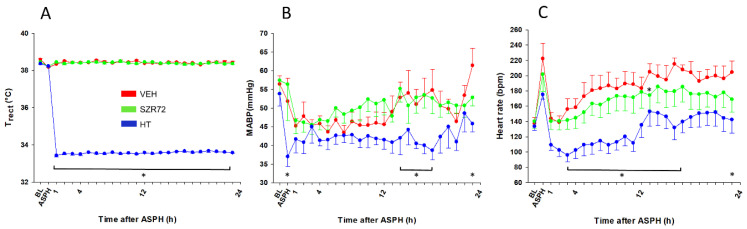
Physiological parameters. (**A**): In the normothermic vehicle-treated (VEH) and SZR72-treated (SZR72) groups, rectal temperature was maintained at 38.5 °C throughout the experiments. The hypothermia-treated (HT) group was also normothermic at baseline (BL) and during the asphyxia (ASPH); however, the rectal temperature reached therapeutic levels at 33.5 °C by 1 h after asphyxia and was maintained at that level for the rest of the observation period. (**B**): mean arterial blood pressure (MABP) was within the normal range for all groups, although it was significantly lower in the HT group at the end of the asphyxia. There was no significant difference between the VEH and the SZR72 groups over the whole observation period, but it tended to be lower in the HT group that reached statistical significance first at 14 h. (**C**): heart rate was elevated by asphyxia from baseline levels, and it remained elevated in the normothermic groups throughout the observation period. There was a tendency for a somewhat smaller heart rate in the SZR72 group; however, there was no significant difference between the groups except at 13 h after asphyxia. As expected, hypothermia significantly reduced the heart rate that was significantly different from the corresponding values of the VEH group at most time points. * *p* < 0.05, significantly different from the corresponding value of the VEH group for all time points in the brackets and also for individual time points. Significant differences from the respective baselines within the groups are not indicated for clarity.

**Figure 3:**
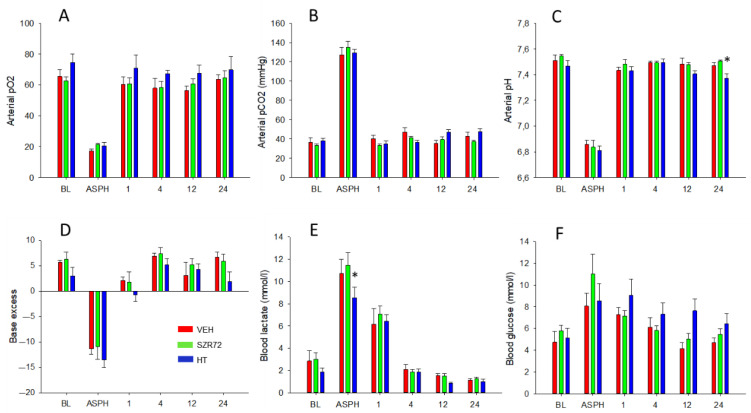
Blood chemistry data. Compared to baseline (BL) levels, asphyxia (ASPH) resulted in marked hypoxia (**A**), hypercapnia (**B**), and acidosis (**C**), the latter showing a robust metabolic component, indicated both by negative base excess (**D**) and lactacidosis (**E**). Blood glucose level elevations during asphyxia were not statistically significant (**F**). Blood gases, pH, and base excess were restored by 1 h after asphyxia. Lactic acid levels were still significantly elevated at 1 h then returned to baseline levels by 4 h. There was no difference among the experimental groups in the asphyxia-induced changes in blood gas parameters; only the increase in lactate levels during asphyxia was somewhat lower in the HT group, although this difference was not detected in base deficit, and lactate levels were virtually identical at 1 h after asphyxia in the three groups. In a similar fashion, the post-asphyxia blood gas parameters were very similar in all three experimental groups throughout the observation period, with a tendency for slightly higher blood sugar levels in the HT group. * *p* < 0.05, significantly different from the corresponding value of the VEH group. Significant differences from the respective baselines within the groups are not indicated for clarity.

The experimental protocol is outlined in Figure 1. Body temperature was kept rigorously in the normothermic range during asphyxia in all groups, then cooling commenced in the HT group, in which target body temperature was achieved within 40–50 min (Figure 2A). Mean arterial blood pressure (MABP) was kept in the normal range in all animals in the observation period (Figure 2B), and heart rate (HR) values were typically lower in the HT group (Figure 2C). Experimental asphyxia resulted in severe hypoxia, hypercapnia, and lactic acidosis that were similar in the experimental groups (Figure 3). Alterations in blood gases were quickly reversed upon reventilation; however, lactate levels were typically still elevated at 1 h after asphyxia and returned to baseline levels at 4 h. Then, throughout the post-asphyxial observation period, blood chemistry data were not significantly different among the treatment groups.

**Figure 4:**
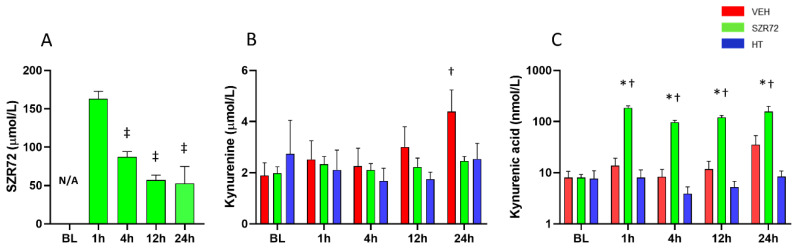
Serum levels of SZR72, kynurenine, and kynurenic acid (KYNA). (**A**): SZR72 levels were highest at 1 h after asphyxia reflecting the effect of bolus drug administration, then they were gradually decreased and stabilized in 50–100 µmol/L range. (**B**): Kynurenine levels were similar among the different groups at baseline (BL), and they were largely unaffected by asphyxia, except there was a statistically significant elevation in the VEH group at 24 h. (**C**): KYNA levels were similar among the different groups at BL, and they were unchanged in the VEH and HT groups after asphyxia, but they were increased 10-fold in the SZR72 group (note the log scale of **C**). *, †, ‡ *p* < 0.05, significantly different from the corresponding value of the VEH group, from the respective baseline value of the group, or from the respective 1 h value of the group, respectively.

SZR72 treatment resulted in the expected large increase in serum SZR72 levels, the continuous SZR72 infusion maintained serum SZR72 concentrations in the ~50–100 µmol/L range (Figure 4A). SZR72 treatment did not affect serum kynurenine but significantly increased serum KYNA levels (Figure 4B,C).

**Figure 5:**
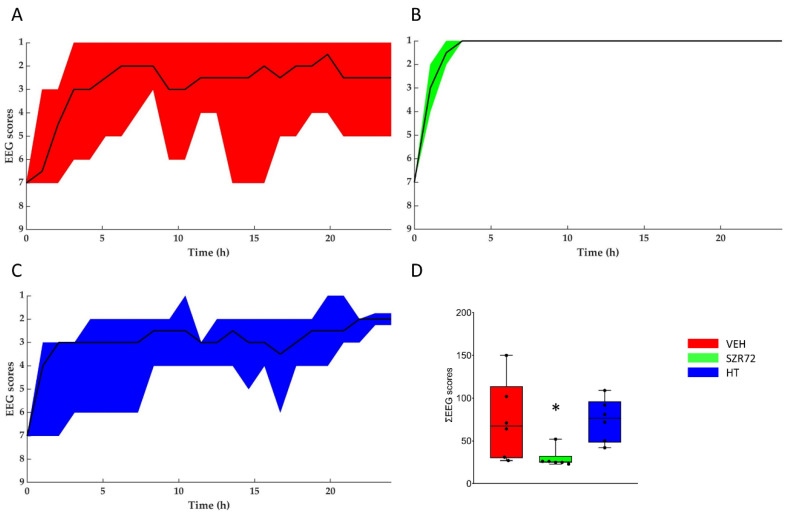
Regeneration of the brain electrical activity shown with the amplitude-based EEG scoring system. (**A–C**): The black lines show the medians, whereas the colors indicate the interquartile ranges. At the onset of reoxygenation after asphyxia, the EEG was flat in all animals (score 7); afterward, it gradually restored to a continuous electrical activity. Quick restoration of EEG activity was conspicuous in the SZR72 group. (**D**): the box plot shows the sum of the EEG scores determined in each hour of the post-insult observation period. The black line is the median, the box shows the interquartile range, the whiskers show the 10th–90th percentiles, and the bullets are the raw data points. The SZR72 group had significantly lower values, in agreement with the quicker and more complete restoration of EEG activity. * *p* < 0.05 significantly different from the VEH group.

**Figure 6:**
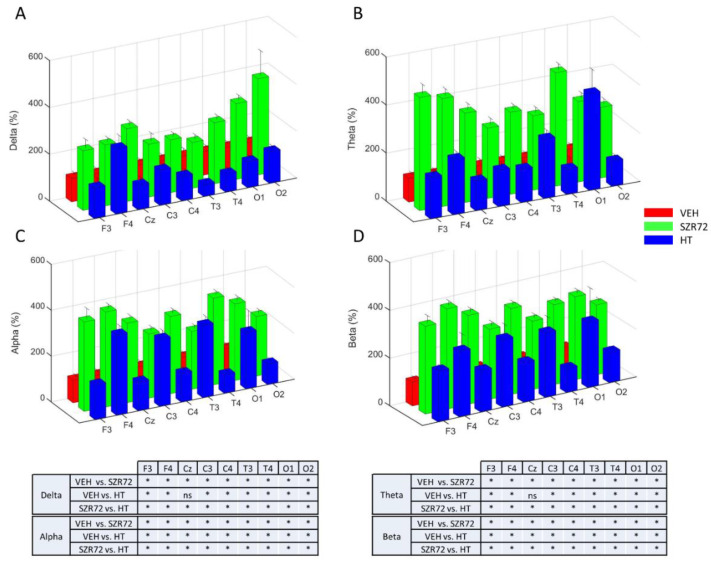
Power spectral density (PSD) analysis of the EEG signal at 24 h after asphyxia. Data are expressed as % of the VEH group (mean ± SD) in the frontal (F), central (C), temporal (T), occipital (O) leads in the respective frequency ranges (**Panels A**–**D**). In all leads and in all frequency ranges, PSDs were consistently much higher in the SZR72 group than in the VEH group. PSDs were also higher in the HT group, compared to VEH, but they were usually lower than in the SZR72 group. Pairwise comparisons are shown in the tables for each frequency range, and leads * *p* < 0.05, n.s. not significant.

**Figure 7:**
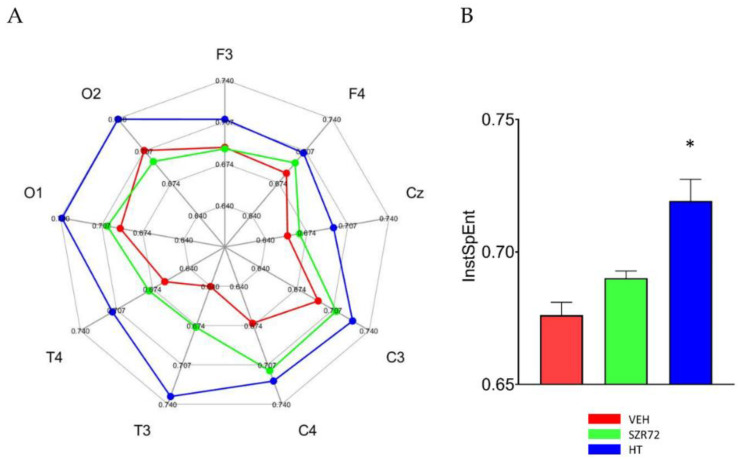
Instantaneous spectral entropy (InstSpEnt) of the EEG signal at 24 h after asphyxia. (**A**): In the spider chart, the average values obtained in the respective EEG leads are shown. In most leads, InstSpEnt values of the VEH group are the smallest representing the least signal complexity, followed by the values in the SZR72 group. In all leads, the HT group unequivocally produced the signal with the highest entropy. (**B**): The bar graph shows the InstSpENt averages of all leads, indicating similar entropy values in the VEH and SZR72 group EEG signal, which are significantly smaller than the values obtained in the HT group. * *p* < 0.05, significantly different from the corresponding value of the VEH group.

**Figure 8:**
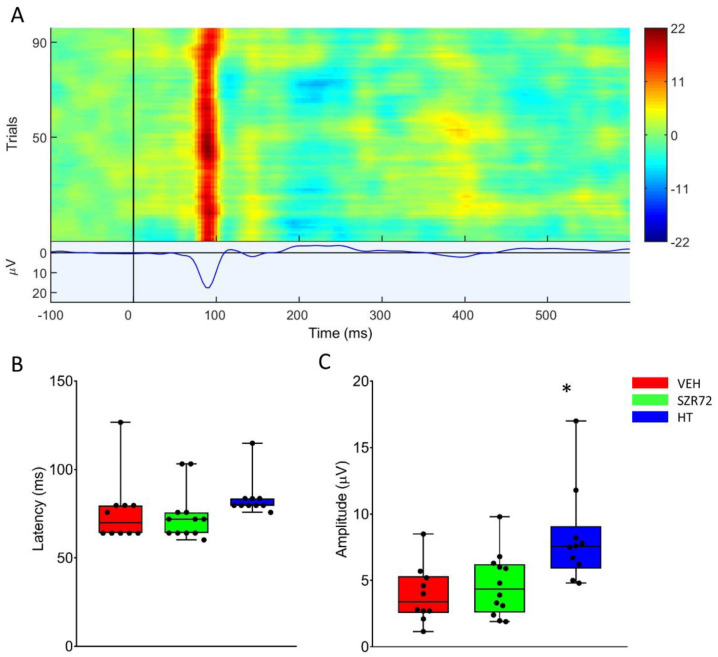
Visual evoked potentials (VEP) evoked at 24 h after asphyxia. (**A**): The heat map shows the responses to the 100 individual light stimuli, constituting the VEP waveform displaying a marked P100 component in a representative record. (**B**): There was no difference among the groups among P100 latency that were unaffected by asphyxia. (**C**): P100 amplitudes in the VEH and SZR72 groups were similar, both decreased from pre-asphyxia baselines. The HT group displayed the highest P100 amplitudes, indicating the best preservation of function. The black line is the median, the box shows the interquartile range, the whiskers show the 10th–90th percentiles, and the bullets are the raw data points. Baseline P100 amplitudes were 8.7 ± 1.7, 8.1 ± 0.6, and 12.0 ± 1.8 µV (mean ± SEM) for VEH, SZR72, and HT groups, respectively. * *p* < 0.05, significantly different from the corresponding value of the VEH group.

Asphyxia resulted in an isoelectric EEG that recovered gradually over the observation period. The return of a continuous high-amplitude EEG was markedly present in all SZR72 animals, and it was completed in 4 h after asphyxia, unlike in the other groups (Figure 5). Furthermore, at 24 h after asphyxia, EEG power spectral density (PSD) analysis revealed that PSD-s virtually in all leads and in all frequency ranges were significantly higher in the SZR72 group, compared to either the VEH or the HT groups (Figure 6). However, instantaneous spectral entropy (InstSpEnt) values reflecting EEG signal complexity showed that, unlike the higher PSD values, the InstSpEnt values in the SZR72 group were quite similar to the VEH group and lower than in the HT group (Figure 7). Concerning VEP, the latency of the P100 component was unaffected by asphyxia, but its amplitude was significantly reduced in the SZR72 and VEH groups but not in the HT group, suggesting a lack of SZR72-induced neuroprotection (Figure 8).

**Figure 9:**
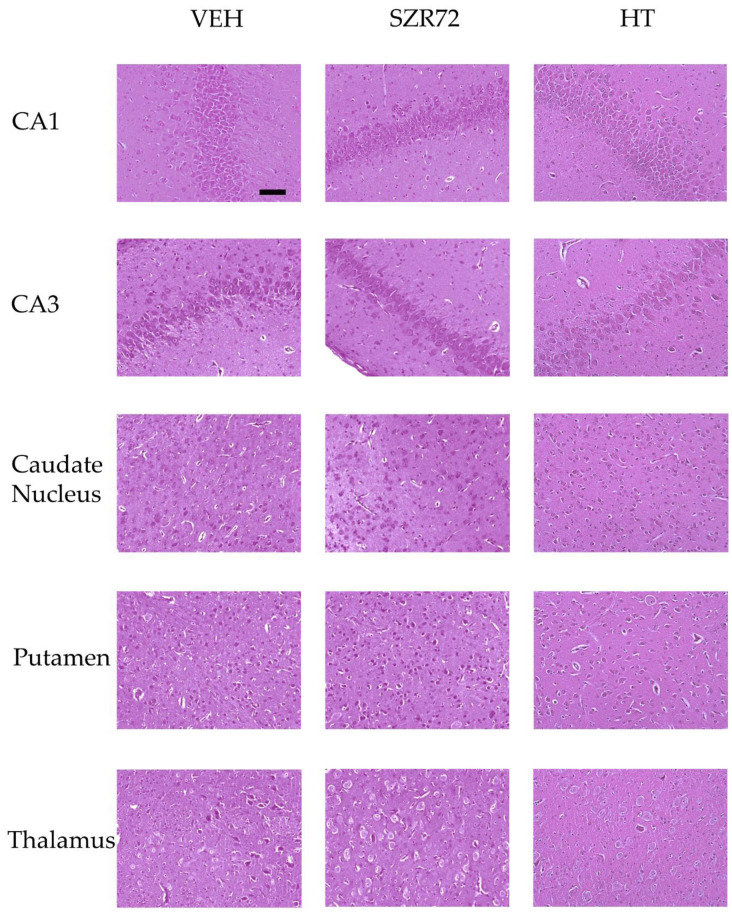
Representative photomicrographs of H&E stained sections from the hippocampal CA1/CA3 subfields, the caudate nucleus, the putamen, and the thalamus. Asphyxia elicited severe neuronal injury that is evident by the large percentage of damaged red neurons in the VEH and the SZR72 group, whereas neuronal damage was markedly less in the HT group. The images were obtained from individuals representing the group median values. Scale bar: 100 μm.

**Figure 10:**
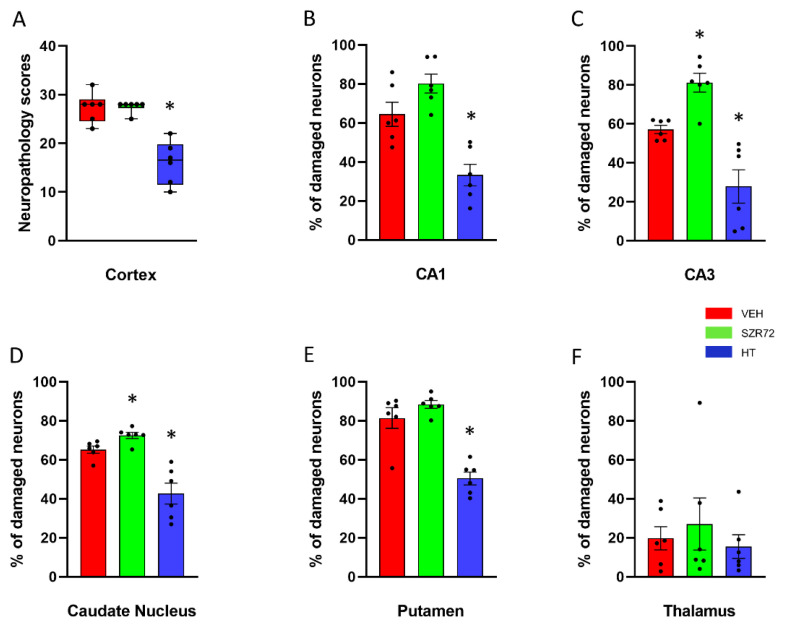
Neuropathology. (**A**): Sum of neuropathological scores determined in the frontal, parietal, occipital, and temporal neocortical areas. The boxes represent the interquartile range, the line within the box represents the median value, and the bullets are the raw data points. Asphyxia induced very similar neocortical damage in the VEH and SZR72 groups; however, neuronal damage was significantly reduced in the HT group. (**B**,**C**): Asphyxia elicited marked neuronal injury in the CA1/CA3 hippocampal subfields in the VEH group that appeared to be even more severe in the SZR72 group: the ratio of damaged neurons was indeed significantly larger in the CA3 in the SZR72, compared to the VEH group. Hypothermia, however, yielded significant neuroprotection in both areas. (**D**,**E**): Similar to the CA3 hippocampal subfield, SZR72 treatment resulted in a slightly but significantly larger neuronal damage, compared to VEH in the caudate nucleus but not in the putamen. HT was significantly neuroprotective in both assessed regions of the basal ganglia. (**F**): In this model, the asphyxia-induced neuronal injury was moderate in the thalamus, and there were no significant differences among the groups. * *p* < 0.05 significantly different from the VEH group. Significant differences between the SZR72 and the HT groups are not shown for clarity.

Neuropathology assessment found marked neuronal injury in the neocortex, the hippocampus, the basal ganglia of the VEH groups; however, neuronal damage in the thalamus was less conspicuous (Figure 9 and Figure 10). The degree and the pattern of damage were very similar in the SZR72 group in all assessed regions; however, in two regions, the hippocampal CA3 subfield and the caudate nucleus the percentage of damaged neurons were slightly but statistically significantly higher in the SZR72 group (Figure 10). Neuronal injury was significantly smaller in all regions vulnerable to asphyxia in the HT group, indicating the potent neuroprotective effect of HT.

## 3. Discussion

The major findings of the present study are the following: (1) we demonstrated that the applied SZR72 treatment successfully elevated blood levels of the KYNA analog drug and KYNA levels themselves; (2) SZR72 administration did not affect significantly the monitored physiological parameters but resulted in robust increases in the post-insult power spectra of virtually all EEG frequency ranges and leads, compared to its vehicle, suggesting a direct neuronal effect of SZR72; and (3) despite its marked electrophysiological effects, SZR72 did not prevent selective neuronal damage in the subacute phase of our HIE model.

The present study was performed using newborn (postnatal day 1; PD1) piglets that are currently one of the best preclinical translational large animal models to study HIE. The gross anatomical features of its gyrencephalic brain, the percent size of the brain compared to its final size at birth, and cerebral metabolic rates of glucose are all very similar in piglets, compared to term human infants [15,16]. Furthermore, there is an important neurodevelopmental analogy: in both species, the so-called neuronal growth spurt occurs at the time of birth unlike in rodents having a postnatal or in macaques having a prenatal-skewed growth spurt [17]. Accordingly, birth asphyxia results in a similar physiological response and in a similar pattern of selective neuronal injury in piglets and humans [16,18,19]. In the piglet model, asphyxia-induced neuronal injury affects predominantly the striatum of the basal ganglia and the cerebral cortex. In these areas, the mechanism of neuronal injury is mainly necrosis that is essentially completed in the striatum [20] and also becomes apparent in the cerebral cortex [21] at 24 h after asphyxia. In accordance with these previous observations, we could also detect the asphyxia-induced severe neuronal injury in all these brain areas in the present study. Although the inflicted hypoxic–ischemic stress was severe, it was not too severe to study experimental neuroprotection. A rather significant portion of neurons was proven yet salvageable shown by the efficacy of hypothermia treatment to mitigate neuronal injury in the “positive control” group of our study in all affected regions, even in the most vulnerable region, the putamen. This neuroprotective effect is in perfect agreement with previous reports on the robust neuroprotective effect of hypothermia in piglets [22,23]. Therefore, the experimental conditions were right to assess the acute neuroprotective efficacy of SZR72 in the present study.

Asphyxia-induced neuronal injury is continuously evolving during HIE development, although it is established to recognize distinct phases mostly based on brain energetics (levels of high energy phosphates and lactate) assessed with magnetic resonance spectroscopy [24]. These include the primary energy failure during asphyxia, followed by energy recovery in the first hour upon reoxygenation/reventilation, followed by the so-called latent phase characterized by near-normal energy levels. The term secondary energy failure describes the onset of a second progressive deterioration of brain energetics persisting for many days [25] caused by neuronal mitochondrial injury accumulated in the preceding phases [24]. The start of a slow gradual restoration of brain energy levels marks the onset of the tertiary phase. The 24 h observation period used in our present study allowed us to follow up HIE development into the secondary energy failure phase. The duration of the latent phase is variable (6–24 h) since it is inversely related to both the severity of hypoxic–ischemic stress and body temperature [26]. Based on the severity of neuronal injury detected in [26] and the present study, the secondary energy failure likely started between 10 and 20 h after asphyxia in the normothermic animals of our study. Indeed, widespread irreversible ultrastructural mitochondrial damage was found at 12 h (but not at 6 h) after asphyxia in a piglet study using very similar asphyxia stress yielding similar levels of neuronal injury, compared to our present study [20]. Therefore, the applied SZR72 treatment over the first 24 h of HIE development in the present study likely spanned the whole latent phase that is widely accepted to be the primary therapeutic window for neuroprotective interventions.

KYNA was found to be an endogenous inhibitor of NMDA receptors [27], triggering intense research that identified KYNA as a competitive NMDA receptor antagonist acting at the strychnine-sensitive glycine binding site of the NMDA receptor in the concentration range of 10–30 µM, whereas in much higher concentrations, it could antagonize the NMDA binding site as well [28,29]. Furthermore, KYNA is supposed to be also a potent inhibitor of the α7 nicotinic acetyl–choline receptors that can also modulate glutamate release from presynaptic terminals [30], although more recently, this effect of KYNA has been questioned [31]. Excessive glutamate release and excitotoxic activation of NMDA receptors are important mechanisms of neuronal injury during the primary energy failure and the reoxygenation/reperfusion phase during HIE development, and pretreatment with the NMDA receptor antagonist MK-801 offered full, while post-hypoxic treatment yielded partial neuroprotection in a rat pup HIE model [32]. However, MK-801 was only partially effective even using a pretreatment protocol in a piglet HIE model [33], and MK-801 did have not only protection but also toxicity in the rat pups as well [34]. Therefore, the use of alternative, endogenous anti-excitotoxic agents are of interest, and systemic administration of KYNA starting before hypoxic–ischemic stress and continuing in the 24 h observation period reduced brain edema [35], and even only post-insult KYNA administration was shown to exert long lasting (assessed at 2 weeks after hypoxic–ischemic stress) neuroprotection [7]. In addition to the anti-excitotoxic mechanism of neuroprotection, other protective mechanisms of KYNA have been proposed, such as a direct antioxidant [36] or an anti-inflammatory effect [37] that are indeed involved in HIE pathophysiology [16,24].

A clear disadvantage of exogenous administration of KYNA is its poor blood–brain barrier permeability [38]. Both studies [7,35] showing KYNA-induced neuroprotection used the Rice–Vannucci HIE model that employs unilateral carotid artery occlusion combined with hypoxia in P7 rat pups, resulting in a focal lesion in which blood–brain barrier integrity is compromised in the early reoxygenation phase [39,40]. In contrast, bona fide asphyxia induced by ventilation with a hypoxic–hypercapnic gas mixture did not deteriorate the blood–brain barrier in P6/P11 rat pups [41]. In our previous piglet study [42], we also observed maintained blood–brain barrier integrity using the same asphyxia model as in [41] and in the current study. We believe that the poor blood–brain barrier permeability of KYNA largely explains the paucity of studies assessing its neuroprotective action in any large animal HIE models. When applied directly onto the cortical surface, thus circumventing the blood–brain barrier, KYNA could antagonize NMDA actions, suggesting its efficacy in the piglet as well [9]. The bioavailability of exogenous kynurenine is better than that of KYNA; however, kynurenine can be metabolized to KYNA and to the neurotoxic quinolinic acid; indeed, exogenous kynurenine was found to worsen neurological outcome in an adult rat stroke model [43].

SZR72 has been developed to produce a KYNA analog with increased bioavailability to explore its neuroprotective potential in various neurological diseases [11]. Indeed, SZR72 was shown to exert antinociceptive effects in preclinical headache models [13,44,45], in a transgenic mouse model of Huntington’s disease [46], and importantly in a four-vessel occlusion rat global cerebral ischemia model [14], in which even a single post-insult SZR72 administration was effective, although a combined pretreatment and repeated post-insult application protocol was clearly superior to that. Importantly, the neuroprotective dose (300 mg/kg) used did not deteriorate cognitive functions assessed with behavioral tests in intact rats or mice [47]. In our present study, we employed only a translationally relevant post-insult SZR72 treatment protocol that resulted in robust elevations in serum SZR72 levels. Serum KYNA levels were also elevated subsequent to SZR72 administration suggesting some minor conversion of SZR72 to KYNA in situ. We cannot know the brain SZR72 levels, but a clear indication of SZR72 passing through the blood–brain barrier in significant amounts is the observed enhancement of neuronal activity shown both by the more rapid restoration of the EEG after asphyxia and the increases in PSD values virtually in all frequency ranges in the SZR72, compared to the VEH group. These differences cannot be explained by any differences in the monitored physiological parameters (MABP, SpO_2_, lactate levels, etc) but only by the direct neuronal action of SZR72. Enhancement of neuronal activity by an anti-glutamatergic agent may be unexpected but not unprecedented: SZR72 was found previously to facilitate CA1 hippocampal long-term potentiation in vivo in rats [48], perhaps through the reported concentration-dependent, KYNA-induced facilitation of α-amino-3-hydroxy-5-methyl-4-isoxazoleproprionic acid (AMPA)-sensitive glutamate receptors [49]. This effect is in concert with a similar, concentration-dependent Janus-faced action of KYNA itself since it was shown to enhance field excitatory postsynaptic potentials in the nanomolar range while dose-dependently inhibiting them in the micromolar concentrations [50]. Despite the marked neuronal effect of SZR72 in the present study, it failed to prevent neuronal damage in any of the assessed regions. Moreover, in the CA3 hippocampal subfield and the caudate nucleus, the neuronal injury was slightly but statistically significantly even more severe. The cause of this negative result is unknown, but several factors may have contributed to this result. First, the observed enhancement of neuronal activity could have increased the energy demands of neurons resulting in critical energy depletion in more neurons. Indeed, in contrast to PSDs, electrophysiological indicators of functional neuronal integrity such as VEP P100 amplitude, or InstSpEnt values did not indicate a better outcome in the SZR72-treated animals. Second, SZR72 in its neuroprotective dose was found to induce hypothermia in freely moving rats by up to 2 °C that could contribute to its neuroprotective effect [51] unless body temperature was rigorously maintained throughout the drug treatment as in our present study. Third, NMDA-receptor-mediated neuronal injury can be enhanced by over-activation of other ion channels such as the acid-sensing ion channel 1a (ASIC1a), indeed, an ASIC1a inhibitor showed an additive neuroprotective effect with MK-801 in a piglet HIE model [33]. Since in our HIE model, severe brain acidosis develops, suggesting strong activation of ASIC channels lasting in the first hour of reventilation after asphyxia [42], perhaps SZR72 could not provide sufficient NMDA-receptor blocking in this time period that might have been critical in determining the neuronal outcome. Recently, new KYNA analogs stemming from SZR72 have been synthesized and proven superior penetration in an in vitro blood–brain barrier model, compared to SZR72 retaining their advantageous biological activity [52]. Thus, despite the negative overall outcome of the present study, it lays the foundation of further studies designed to evaluate the neuroprotective effects of these new analogs to combat HIE.

## 4. Materials and Methods

### 4.1. Animals

The experimental procedures were reviewed and approved by the Hungarian National Scientific Ethical Committee on Animal Experimentation (ÁTET), and then the necessary permit to obtain the animals was issued by the National Food Chain Safety and Animal Health Directorate of Csongrád county, Hungary (permit nr: XIV./1414/2015). The procedures were performed according to the guidelines of the Scientific Committee of Animal Experimentation of the Hungarian Academy of Sciences (updated Law and Regulations on Animal Protection: 40/2013. (II. 14.) Gov. of Hungary), following the EU Directive 2010/63/EU on the protection of animals used for scientific purposes and reported in compliance with the ARRIVE guidelines.

Newborn (PD1) male Landrace piglets (*n* = 18, weighing between: 1.5 and 2.5 kg) were obtained from a local company (Pigmark Ltd., Co., Szeged, Hungary) and delivered to the laboratory on the morning of the experiments. The animals were anesthetized with an intraperitoneal injection of sodium thiopental (45 mg/kg; Sandoz, Kundl, Austria), then were placed on a servo-controlled heating pad (Blanketrol III, Cincinnati SUB-zero, Cincinnati, OH, USA) to keep their rectal temperature in the physiological range (38.5 ± 0.5 °C). The skin was disinfected, and the animals were intubated through a tracheotomy, then mechanically ventilated by a pressure-controlled small animal respirator with warmed, humidified medical air (21% O_2_, balance N_2_) at a frequency of 30–35 breaths/min, applying peak inspiratory pressure = 120–135 mmH_2_O to keep blood gases and oxygen saturation within the physiological range. The right carotid artery and femoral vein were cannulated with catheters under aseptic conditions to monitor MABP to take arterial blood samples and administer drugs and fluids, respectively. The wounds were then closed. To maintain anesthesia/analgesia, the animals were given iv. a bolus injection of morphine (100 μg/kg; Teva, Petach Tikva, Israel) and midazolam (250 μg/kg; Torrex Pharma, Vienna, Austria), followed by continuous infusion (morphine 10 μg/kg/h, midazolam 250 μg/kg/h) and were supplemented with fluids (5% glucose, 0.45% NaCl 3–5 mL/kg/h). Prophylactic antibiotics were given intravenously (penicillin: 50 mg/kg, Teva, Petah Tikva, Israel, and gentamicin: 2.5 mg/kg, Sanofi, Paris, France) every 12 h. Dopamine (5–20 µg/kg/min; Admeda Arzneimittel GmbH, Nienwohld, Germany) infusion was given to maintain MABP above 40 mmHg in some animals (*n* = 3, 1, 3 in the VEH, SZR72, HT groups, respectively). The average total dopamine use was 2.1 ± 0.7, 6.5, and 4.6 ± 2.6 mg/kg in the respective groups. The instrumented animals were placed in a prone position into a neonatal incubator (SPC 78-1; Narco Air-Shields, Inc., Hatboro, PA, USA). Oxygen saturation (SpO2), MABP, HR, and electrocardiogram (ECG) were continuously monitored by using EDAN Im8 Vet Monitor (Edan Instruments Inc., Shekou, Nanshan, Shenzhen, China) and recorded online. Arterial blood samples (~300 μL) were analyzed for pH, pCO_2_, pO_2_, along with blood sugar and lactate levels with an epoc^®^ Blood Analysis System (Epocal Inc., Ottawa, ON, Canada) at baseline, at the end of asphyxia and then at selected intervals up to 24 h (Figure 1) to keep blood gas values in the physiological range during the survival period. The urinary bladder was tapped by a suprapubic puncture at 12 h after asphyxia. At the end of the 24 h observation period, both carotid arteries of the anesthetized animals were catheterized in the distal direction, the animals were euthanized with pentobarbital sodium (300 mg, Release^®^; Wirtschaftsgenossenschaft Deutscher Tierärzte eG, Garbsen, Germany), and then the brains were perfused with cold (4 °C) physiological saline. The brains were gently removed from the skull and the intact right hemispheres were immersion-fixed in 4 °C, 4% paraformaldehyde solution and further processed for histology.

### 4.2. Experimental Protocol

The experimental protocol is shown in Figure 1. Baseline physiological parameters were obtained following a ~1 h stabilization period after surgery. Animals were assigned to one of the following three groups: (1) VEH (*n* = 6), (2) SZR72 (*n* = 6), and (3) HT (*n* = 6). In each group, experimental asphyxia was induced by ventilation with a hypoxic–hypercapnic gas mixture containing 6% O_2_ and 20% CO_2_ for 20 min while the respiratory rate was reduced from 30 to 15 breaths/min, and intravenous glucose administration was suspended. Reventilation commenced with medical air (room air) throughout the 24 h observation period (respiration rate; RR: 30 1/min). In the VEH and the SZR72 groups, vehicle/drug treatment started 5 min after the completion of asphyxia (see details at 4.3). In the HT group, body cooling started simultaneously with reventilation using the servo-controlled heating pad. The rectal temperature reached 33.5 °C in 40–50 min and was maintained throughout the experiment.

### 4.3. SZR72 Treatment

SZR72 was synthesized in the Institute of Pharmaceutical Chemistry, University of Szeged [11]. Most previous in vivo rodent studies assessing CNS function used SZR72 in a 300 mg/kg (~1 mmol/kg; ip) bolus dose that was repeated typically 2 times every 24 h [12,13,14]. Equivalent dose calculation for newborn piglets based on [53] and [54] resulted in a 170 mg/kg bolus dose that was used in the present study. SZR72 was dissolved in physiological saline to obtain a 40 mg/mL solution, and then the pH of the solution was adjusted to 7.4 using 1 M NaOH. SZR72 administration started 5 min after the completion of asphyxia; 170 mg/kg was given as a fast iv infusion in 5 min. The bolus was followed by a continuous infusion of SZR72 (170 mg/kg/12 h) throughout the 24 h observation period. The vehicle group received a physiological saline infusion.

### 4.4. Electroencephalography (EEG) and Visual Evoked Potential (VEP)

All EEG/VEP processes were performed according to the American Clinical Neurophysiology Society’s guidelines [55,56]. EEG recordings were taken via subcutaneously inserted silver scalp electrodes using a previously published eight-lead (fronto–parieto–centro–occipital) montage and a 256 Hz sampling rate [57]. The electrode impedance was regularly checked to stay below 5 kΩ. The EEG signals were amplified, recorded, and visualized with the Nicolet EEG (Natus Neurology, Middleton, WI, USA) and software [58]. The recorded EEG was scored based on background EEG amplitudes using a previously published scoring system [57,59] based on [60]. Briefly, continuous high-amplitude (>25 µV) activity is scored 1, while an isoelectric trace is scored 7. VEP was evoked with stroboscope-generated 1 Hz flashes (10 trains of 10 stimuli each with 10 s intervals between the trains). VEP waveforms were determined by averaging the 100 trials. The amplitude and latency of the P100 component were determined, and their grand mean averages were calculated.

### 4.5. EEG Spectral Analysis

All electrophysiology data were analyzed in a MATLAB environment with custom-written scripts, built-in functions, and the EEGlab toolbox [61]. The broadband EEG signals were band-pass filtered (1–30 Hz) and decomposed into the four main physiological frequency ranges (delta (1–4 Hz), theta (4–8 Hz), alpha (8–13 Hz), and beta (13–30 Hz)). We used fast Fourier transform-based (FFT) calculations for determining the PSDs, applying a Gaussian window on the signals. The averaged and summed PSDs were normalized to the baseline [62]. The InstSpEnt was calculated as published previously [57].

### 4.6. Serum Sample Processing for Determination of Kynurenine, KYNA, and SZR72 Levels

Arterial blood samples (1 mL) at selected time intervals (Figure 1) were collected and allowed to clot, followed by centrifugation (4 °C; 10 min; 13,000 rpm) to obtain serum samples that were kept at −80 °C until analysis. For targeted analytical measurements, LC–MS grade solvents were used in all cases. The method for the preparation of serum samples prior to the analysis of endogenous kynurenine and KYNA was the following. First, 50 µL serum was spiked with 7 µL internal standard (IS) solution containing SZR73 (0.5 µM) in water/methanol (50/50 *v*/*v*) [52], and 5 µL water/methanol (50/50 *v*/*v*) solution, then 600 µL ice-cold acetone was added in a 1.7 mL microcentrifuge tube (Corning-Costar 3620, Corning Inc., Corning, NY, USA). The sample was vortex mixed for 15 s, after shaking for 10 min at room temperature the sample was centrifuged at 15,000 rpm for 15 min at 4 °C degrees (Hettich 320R, Hettich Gmbh, Tuttlingen, Germany). The 600 µL of the upper layer was transferred to a microcentrifuge tube and evaporated to dryness under nitrogen at ambient temperature (MD 200, Allsheng Instruments Ltd., Hangzhou, China). For analysis, the dried extracts were dissolved in 50 µL of H_2_O/MeOH/NH3 (90/10/0.1 *v*/*v*/*v*%), vortex mixed for 15 s, centrifuged at 15,000 rpm for 15 min at 22 °C degrees, and the upper layer transferred to a 250 µL conical insert. For calibration samples, 50 µL pulled control serum sample was spiked with 7 µL IS, then 5 µL given calibration mix containing kynurenine and KYNA in water/methanol (50/50 *v*/*v*) solution, then 5 µL IS, and then 600 µL ice-cold acetone was added in a microcentrifuge tube. Then, the above-described sample preparation protocol was applied. The calibration points were the followings: 0, 2.79, 2.89, 2.99, 3.29, 4.79, and 6.79 µM for kynurenine, and 0, 23.98, 24.98, 25.98, 28.98, 33.98, 43.98, 63.98, 123.98, and 523.98 nM for KYNA.

For the analysis of SZR72, the applied sample preparation procedure was slightly modified. In brief, 10 µL serum was spiked with 7 µL IS solution containing SZR73 (10 µM) in water/methanol (50/50 *v*/*v*) and 10 µL water/methanol (50/50 *v*/*v*) solution, then 600 µL ice-cold acetone was added in a 1.7 mL microcentrifuge tube (Corning-Costar 3620, USA). The parameters of vortex mixing, shaking, centrifugation, collection of upper phase, and evaporation were the same as used for kynurenine/KYNA analysis. The dried extracts were dissolved in 500 µL of H_2_O/MeOH/NH3 (90/10/0.1 *v*/*v*/*v*%), vortex mixed for 15 s, centrifuged at 15,000 rpm for 15 min at 22 °C degrees, and the upper layer transferred to a 250 µL conical insert. In the case of calibration samples, calibration points were set to 0, 5, 10, 50, 100, and 200 µM for SZR72.

### 4.7. Ultrahigh Performance Liquid Chromatography Coupled to Tandem Mass Spectrometry (UHPLC–MS/MS) Parameters for Quantitative Analysis of Kynurenine, KYNA, and SZR72

The targeted UHPLC–MS/MS analysis was performed on a UHPLC (Nexera, Shimadzu, Kyoto, Japan) coupled to a triple quadrupole mass spectrometer (TSQ Fortis, ThermoFisher Scientific, Waltham, MA, USA) with an OptaMax NG heated electrospray ionization source (ThermoFisher). The UHPLC was controlled using the manufacturer’s software [63], and data were acquired and evaluated in accordance with [64].

The developed targeted UHPLC–MS/MS method was as follows: ACQUITY UPLC HSS C18 column (100 × 2.1 mm, 1.8 μm, 100 Å, Waters, Milford, MA, USA). The UHPLC mobile phase A consisted of 0.1% formic acid solution, and mobile phase B was composed of methanol with 0.1% *v*/*v* formic acid. The gradient program started with 0.4 mL/min flow rate and 10% B, ramped to 100% B in 3 min and hold for 0.2 min, then flow rate ramped to 0.5 mL/min within 0.1 min, 100% B held for another 1.7 min, returned to 10% B within 0.1 min, held for 4 min, returned to 0.4 mL/min flow rate within 0.1 min, and the initial condition held for 0.9 min. The column temperature was maintained at 50 °C, the autosampler temperature was 15 °C, and 10 µL samples were used for analysis. The injector needle was washed with 2-propanol/MeOH/H_2_O/FA (70/25/5/0.1, *v*/*v*/*v*/*v*%) solution after each injection.

The mass spectrometer was operating in positive scheduled multiple reaction monitoring modes using a heated electrospray ionization source (H-ESI). The instrument settings were as follows: capillary temperature 300 °C, vaporizer temperature 350 °C, spray voltage 3.9 kV, sheath gas flow 20, sweep gas flow 1, and auxiliary gas flow 5 arbitrary unit. The data acquisition was performed in the selected reaction monitoring mode with both Q1 and Q3 resolution FWHM at 0.7. The proper quantifier and qualifier ions of given protonated precursor ions of each analyte with the related collision energies, tube lens voltages were determined by the flow injection method. In order to decrease the contamination of the mass spectrometer, the eluate was passed into the H-ESI source only in the time range of 1.0–3.2 min. In the remaining time, the source was rinsed with acetonitrile/water solution (90/10, *v*/*v*) at a flow rate of 0.2 mL/min by an Agilent 1100 isocratic pump (Agilent Technologies Inc., Waldbronn, Frankfurt, Germany). The main UHPLC–MS/MS parameters are summarized in Table 1.

The standard addition method with calibration points was used for the quantification of the endogenous kynurenine and KYNA, while a six-point curve of the external calibration was applied for the quantitative evaluation of exogenous SZR72. In all cases, the evaluation was based on quantifier ion of analyte/quantifier ion of SZR73 peak area ratios vs. concentration.

### 4.8. Histology

Tissue samples were dissected from the frontal, temporal, parietal, occipital lobes as well as the hippocampus CA1/CA3, thalamus, putamen, and nucleus caudatus areas, paraffin-embedded and 4-μm sections were produced using a microtome (Leica Microsystems, Wetzlar, Germany) and mounted on silanized slides. Hematoxylin–eosin staining was performed to evaluate the extent of neuronal damage in the subcortical fields, which was assessed with manual cell counting by two independent observers in nonoverlapping areas using ImageJ [65]. Damaged neurons were identified using the major hallmarks of dark eosinophilic cytosol, as well as pyknotic or disrupted nuclei by a researcher blinded to the experimental groups. In the hippocampus and subcortical brain regions, neuronal injury was expressed as the percentage of damaged neurons. In the cerebral cortex, neuropathology scores were determined (0–9), as described previously [57,59]. Briefly, the pattern of neuronal injury (none < scattered < grouped/laminar < panlaminar) was evaluated in 40 nonoverlapping fields of vision under 20× magnification with light microscopy (Leica Microsystems, Wetzlar, Germany) in each cortical region. Then, neuropathology scores (0–9) were given to each cortical region based on the abundance of the most severe injury pattern. Thus, higher scores represent increasingly severe neuronal damage.

### 4.9. Statistical Analysis

Statistics were conducted using the software packages [66,67,68]. Parametric data are presented as mean ± SEM, while nonparametric data as the median and the interquartile range unless stated otherwise. *p* values < 0.05 were considered statistically significant. Differences in core temperature, MABP, HR, pO_2_, pCO_2_, pH, base excess, lactate, and glucose were compared among the three groups at each time point by two-way repeated-measures ANOVA. Pairwise comparisons were then performed using the Student–Newman–Keuls (SNK) post hoc test. Serum levels of SZR72 at different time points were analyzed using one-way repeated-measures ANOVA, followed by the SNK post hoc test. Serum kynurenine and KYNA levels were analyzed among the three groups at respective time points by two-way repeated-measures ANOVA followed by the SNK post hoc test. Neuropathology data were analyzed using one-way ANOVA or Kruskal–Wallis analysis of ranks, followed by the SNK post hoc test for pairwise comparisons. The electrophysiological data (InstSpEnt, PSD, VEP) are presented as mean ± SD. InstSpEnt data were compared with one-way repeated-measures ANOVA, whereas PSD and VEP data were compared with two-way repeated-measures ANOVA. Pairwise comparisons in each case were performed using Tukey’s post hoc test.

## 5. Conclusions

In a preclinical large animal HIE model, experimental asphyxia elicited severe neuronal injury that could be significantly ameliorated by therapeutic hypothermia. The KYNA analog SZR72, administered using a translationally relevant post-insult protocol unequivocally enhanced background EEG activity clearly indicating blood–brain barrier penetration and direct neuronal effects of the drug. However, SZR72 failed to improve functional measures of neuronal activity and to mitigate neuronal damage, unlike hypothermia. Our results suggest that the use of exogenous KYNA analogs with higher neuroprotective/less atypical neuronal actions may be feasible in the management of HIE, warranting further preclinical research.

## Figures and Tables

**Table 1:** Main UHPLC–MS/MS parameters of MRM transitions of each analyte.

Compound	Retention Time (min)	Retention Time Window (min)	Precursor Ion (m/z)	Type of Product Ion	Product Ion (m/z)	Collision Energy (eV)	RF Lens (V)
Kynurenine	1.45	1.0	209.2	quantifier	192.1	10	52
Kynurenine	1.45	1.0	209.2	qualifier	146.0	19	52
SZR72	2.43	0.4	260.1	quantifier	215.0	18	75
SZR72	2.43	0.4	260.1	qualifier	144.0	32	75
SZR73 (IS)	2.57	0.4	274.2	quantifier	144.0	37	65
SZR73 (IS)	2.57	0.4	274.2	qualifier	229.0	17	65
KYNA	2.84	0.45	190.1	quantifier	144.0	19	55
KYNA	2.84	0.45	190.1	qualifier	172.0	13	55

## Abbreviations

ECG	electrocardiogram
EEG	electroencephalogram
HIE	Hypoxic–ischemic encephalopathy
HT	hypothermia-treated group
HR	heart rate
InstSpEnt	instantaneous spectral entropy
KYNA	kynurenic acid
MABP	mean arterial blood pressure
PD	postnatal day
PSD	power spectral density
RR	respiration rate
SpO2	oxygen saturation
SZR72	SZR72-treated group
VEH	vehicle-treated group
VEP	visual evoked potential
